# Common computations for metacognition and meta-metacognition

**DOI:** 10.1093/nc/niad023

**Published:** 2023-11-07

**Authors:** Yunxuan Zheng, Samuel Recht, Dobromir Rahnev

**Affiliations:** School of Psychology, Georgia Institute of Technology, Atlanta, GA 30332, United States; Department of Experimental Psychology, University of Oxford, Oxford OX3 7JX, United Kingdom; School of Psychology, Georgia Institute of Technology, Atlanta, GA 30332, United States

**Keywords:** metacognition, meta-metacognition, confidence, perceptual decision-making

## Abstract

Recent evidence shows that people have the meta-metacognitive ability to evaluate their metacognitive judgments of confidence. However, it is unclear whether meta-metacognitive judgments are made by a different system and rely on a separate set of computations compared to metacognitive judgments. To address this question, we asked participants (*N* = 36) to perform a perceptual decision-making task and provide (i) an object-level, Type-1 response about the identity of the stimulus; (ii) a metacognitive, Type-2 response (low/high) regarding their confidence in their Type-1 decision; and (iii) a meta-metacognitive, Type-3 response (low/high) regarding the quality of their Type-2 rating. We found strong evidence for the existence of Type-3, meta-metacognitive ability. In a separate condition, participants performed an identical task with only a Type-1 response followed by a Type-2 response given on a 4-point scale. We found that the two conditions produced equivalent results such that the combination of binary Type-2 and binary Type-3 responses acts similar to a 4-point Type-2 response. Critically, while Type-2 evaluations were subject to metacognitive noise, Type-3 judgments were made at no additional cost. These results suggest that it is unlikely that there is a distinction between Type-2 and Type-3 systems (metacognition and meta-metacognition) in perceptual decision-making and, instead, a single system can be flexibly adapted to produce both Type-2 and Type-3 evaluations recursively.

## 1 Introduction

The ability to reflect on one’s own cognitive processes is known as metacognition (Nelson and Narens 1990). Metacognition is suggested as an evolutionary advantage of human beings (Shea et al. 2014). In the context of perception, empirical evidence has indicated that human metacognition is dependent on the activity of the prefrontal cortex (Fleming et al. 2010, Morales et al. 2018, Shekhar and Rahnev 2018, Zheng et al. 2021) and can guide learning (Guggenmos et al. 2016), cognitive offloading (Gilbert et al. 2020), information seeking (Desender et al. 2018), and social interactions (Bahrami et al. 2010, Pescetelli and Yeung 2021). Metacognitive deficits have also been associated with a wide range of psychiatric symptoms (Zalla et al. 2015, Rouault et al. 2018, Seow et al. 2021, Zheng et al. 2022).

A recent paper demonstrated that humans have the capacity to perform repeated hierarchical evaluations of their judgments up to at least fourth-order judgments (Recht et al. 2022). This finding was recently replicated by a different group of researchers (Sherman and Seth 2023). Here, we follow up on this work to shed light on whether third-order judgments (meta-metacognition) are produced by a different system compared to second-order judgments (metacognition).

The possible distinction between metacognition and meta-metacognition has parallels with a related distinction between cognition and metacognition. One dominant view postulates that there are different, though perhaps interrelated, first- and second-order systems that produce object-level (Type-1) and metacognitive (Type-2) decisions. This view was popularized by Nelson and Narens (1990) who postulated that cognitive processes are split into object-level and meta-level processes. While Nelson and Narens were theorizing in the context of memory, their conceptualization has been very popular in the context of perception too. For example, one influential recent model of perceptual metacognition postulates the existence of a higher-order metacognitive system that is distinct but interrelated to a lower-order decision-making system (Fleming and Daw 2017). An alternative, and perhaps equally popular, view postulates that a single system utilizes the exactly same underlying sensory evidence to compute both cognitive and metacognitive judgments (Kiani and Shadlen 2009, Rahnev et al. 2012, Ratcliff and Starns 2013, Pouget et al. 2016). For example, in a standard signal detection theory framework, both the primary decision and the metacognitive judgment of confidence are made using the same mechanisms and based on the same evidence (Green and Swets 1966, Macmillan and Creelman 2004).

**Figure 1.**
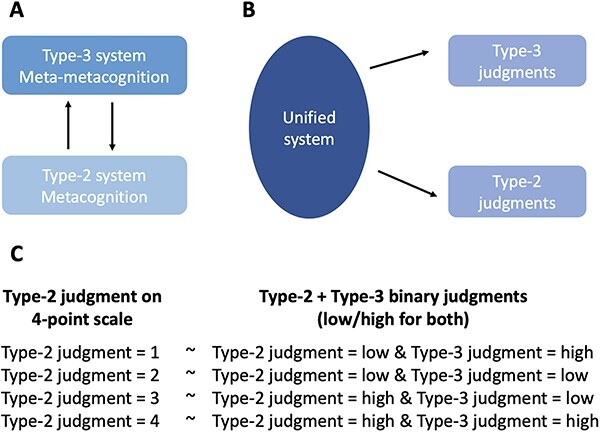
Separate systems vs. a unified system for Type-2 and Type-3 judgments. (a) Depiction of distinct systems for Type-2 metacognition and Type-3 meta-metacognition. (b) Depiction of a single and unified system for Type-2 and Type-3 judgments. (c) A mapping between Type-2 metacognitive judgments given on a 4-point scale and a combination of binary Type-2 metacognitive judgment followed by a binary Type-3 meta-metacognitive judgment

Similar to this debate, there are two broad possibilities about how metacognition and meta-metacognition judgments are made. On one account, we can expect a Type-3 meta-metacognitive judgment to be produced by a different system than a Type-2 metacognitive judgment (Fig. 1a). This distinction would parallel the distinction between Type-2 metacognitive judgments and Type-1 object-level judgments. On a different account, however, the Type-3 and Type-2 judgments would be generated by the same system using the same evidence (Fig. 1b). This possibility would parallel the view that Type-2 and Type-1 judgments are also made by the same system.

In the current study, we explored whether Type-2 and Type-3 judgments are likely to be produced by the same or different systems. Participants completed a simple perceptual decision-making task, followed by either a single Type-2 confidence rating given on a 4-point scale (“Type-2-only” condition) or a combination of a binary (low/high) Type-2 metacognitive rating and a binary (low/high) Type-3 meta-metacognitive rating (“Type-2/Type-3” condition). Critically, each combination of binary Type-2 and binary Type-3 ratings corresponds to a unique Type-2 rating given on a 4-point scale (Fig. 1c). For instance, a confidence rating of 4 on a 4-point Type-2 scale represents that a participant has very high confidence in making a correct decision, which is equivalent to giving high confidence on a 2-point Type-2 scale first followed by a high certainty rating on a 2-point Type-3 scale. Similarly, a confidence rating of 1 on a 4-point Type-2 scale represents that a participant has very low confidence in making a correct decision, which is equivalent to giving low confidence on a 2-point Type-2 scale first followed by a high certainty rating on a 2-point Type-3 scale (i.e. the person is very sure in their low confidence response). Finally, confidence ratings of 2 and 3 correspond to a lack of certainty about whether confidence should be low or high and thus map onto a low Type-3 rating.

We reasoned that if the same system is making both the Type-2 and Type-3 responses, the same behavioral effects would emerge for the corresponding responses in the Type-2-only and the Type-2/Type-3 conditions. Conversely, if there are separate Type-2 and Type-3 systems, different behavioral effects would emerge for the corresponding responses in the two conditions. Our results revealed equivalent effects for corresponding responses between the two conditions across a number of measures, suggesting common computations for metacognition and meta-metacognition and a potentially unified system for Type-2 and Type-3 perceptual judgments.

## Materials and Methods

### 2.1 Participants

Forty adult participants (age: 34.3 ± 11.3 years) were recruited from the Prolific online research platform and were compensated $8 US dollars. All participants reported normal or corrected-to-normal vision, had English as the first language, and had obtained more than 95% approval rate at the Prolific website. Experimental procedures were approved by the Georgia Institute of Technology Institutional Review Board, and written informed consent was provided at the beginning of the experiment to each participant.

To ensure data quality, we excluded participants who had overall decision accuracy below 55% (one participant excluded) or made the same Type-1, Type-2, or Type-3 rating in more than 90% of trials (three participants excluded). Therefore, the final analyses were conducted on data from 36 participants. For each remaining participant, we further excluded trials with response time longer than 3 s (0.5% of all trials were excluded).

### 2.2 Procedure

**Figure 2.**
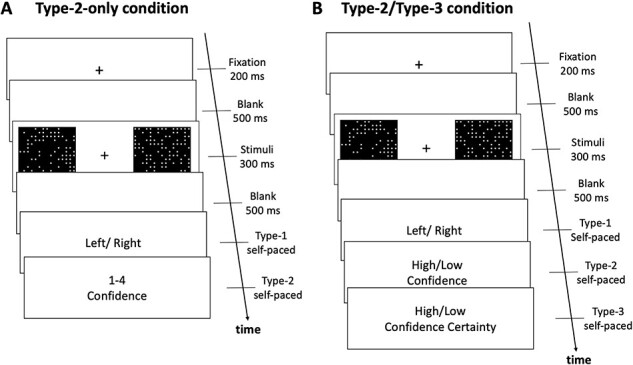
Task paradigm. (a) Type-2-only condition. Participants judged which of two squares contained more dots. On each trial, participants indicated their decision confidence (metacognitive rating) using a 4-point scale. (b) Type-2/Type-3 condition. Participants completed the same dot task. However, instead of giving confidence on a 4-point scale, they provided a confidence rating (Type-2 judgment) on a 2-point scale and then a meta-metacognitive rating (Type-3 judgments) again on a 2-point scale (low/high)

Participants completed a simple perceptual decision-making task where they indicated which of two black squares contained a higher number of white dots (Fig. 2). The two squares were presented on the left and right side of the screen for 300 ms, after a 200 ms fixation and a 500 ms blank screen. One square contained 100 dots, and the other contained 85 dots. The location of white dots in each square was randomly chosen on each trial from an imaginary 15 × 15 grid. The position (left or right) of the square containing more dots was also randomized on each trial.

Each participant completed trials from two different conditions. In the “Type-2-only” condition, participants indicated their perceptual decision (Type-1 judgment) and then rated their decision confidence (Type-2 judgment) on a scale from 1 (“very low confidence”) to 4 (“very high confidence”; Fig. 2a). In the “Type-2/Type-3” condition, participants indicated their perceptual decision (Type-1 judgment) followed by binary Type-2 and Type-3 judgments (Fig. 2b). The Type-3 question was dependent on the Type-2 response. On the one hand, if a participant reported high confidence, the Type-3 question was phrased as follows: “You reported high confidence. Are you absolutely confident, i.e. knowing the correct answer for certain, or do you just have a strong feeling that you are correct this time?” On the other hand, if a participant reported low confidence, the Type-3 question was phrased as follows: “You reported low confidence. Are you absolutely not confident, i.e. no idea which answer is correct, or do you have at least an inkling about the correct answer?” In both cases, participants reported their Type-3 judgment as low (i.e. low certainty in the appropriateness of the Type-2 confidence rating) vs. high (i.e. high certainty in the appropriateness of the Type-2 confidence rating). This wording may have been slightly confusing in situations where a participant made an impulsive response and was very sure in making a mistake. Nevertheless, it was made clear to participants that their Type-3 response is conditional on both the Type-1 and Type-2 responses, so they should select high Type-3 certainty in their low confidence when they think that they have made a wrong decision. In any case, such situations were likely to be rare and we also removed all trials with extremely short Type-1 decision reaction time (RT), which should have removed most impulsive responses. Participants had unlimited time to give the Type-1, Type-2, and Type-3 responses.

Participants completed 400 trials from one condition, followed by 400 trials of the other condition. The order of the two conditions was pseudo-randomly assigned such that 20 participants completed the Type-2/Type-3 condition first and the remaining 20 participants completed the Type-2-only condition first. We chose not to interleave the two conditions on a trial-by-trial basis to avoid the high cognitive demand associated with constantly switching between two modes of responding. In the analyses, we combined both groups together, but in addition also confirmed that all results remain the same when each group is analyzed separately. Each condition was organized in 10 blocks of 40 trials, with participants allowed to take breaks at the end of each block. To ensure the quality of online data, we included an attention check at the end of each block. The attention checks were normal trials, but participants were instructed to choose always right. No participant failed at more than two of the five attention checks.

The experiment was programmed using jsPsych library (Version 5.0.3; de Leeuw 2015). To ensure that the stimulus size was similar across all participants, we adopted an established calibration procedure in our laboratory (Bang et al. 2019) where we asked participants to position the screen at an arm’s distance (∼60 cm) and adjust the size of a credit card displayed on the computer screen to match the dimensions to the actual object in real life.

### 2.3 Data analysis

To determine each participant’s Type-1 task performance and degree of response bias on each condition, we computed the signal detection theory parameter decision sensitivity (*d*') (Green and Swets 1966, Macmillan and Creelman 2004) for each decision and confidence criterion as follows:


$$d^{\prime}= {\phi ^{ - 1}}\left( {{\mathrm{hit}}\,{\mathrm{rate}}} \right) - {\phi ^{ - 1}}\left( {{\mathrm{false}}\,\,{\mathrm{alarm}}\,\,{\mathrm{rate}}} \right),$$

where ${\phi ^{ - 1}}$ is the inverse of the cumulative standard normal distribution that transforms hit rate and false-alarm rate into *z* scores.

To quantify participants’ metacognitive ability when they reported high and low Type-3 certainties, we applied the method developed by Maniscalco and Lau (2012). The method first computes each participant’s metacognitive sensitivity (meta-*d*') that quantifies how well confidence ratings discriminate between Type-1 correct and incorrect responses. To further control for the influence of Type-1 task performance, we finally computed metacognitive efficiency, meta-*d*'/*d*' or Mratio, to represent the participant’s metacognitive ability for a given Type-3 rating.

We also computed average Type-1 accuracy and decision RT for each Type-2 confidence level and compared them across Type-2 levels and across the two task conditions. Note that we also calculated the trial frequency (i.e. the number of trials) for each of the eight possible responses (e.g. left decision with confidence 4 or right decision with confidence 1; see the Supplementary Material). We did not examine the response times associated with the Type-2 and Type-3 decisions because they are not commensurate between the two conditions. Specifically, the Type-2-only condition involves two button presses (i.e. the Type-1 and Type-2 judgments), while the Type-2/Type-3 condition involves three button presses, making it difficult to compare the RTs for anything other than the first button press.

To control for the correlated participants’ error within the repeated conditions, we used linear mixed-effect models (LLMs) with the participant as a random effect to investigate the between-task similarity or difference in terms of Mratio, Type-1 accuracy, Type-1 decision RT, and the *d*' dependence on the decision/confidence criterion used for its computation.

## 3 Results

We investigated whether there are different systems for computing Type-2 metacognitive and Type-3 meta-metacognitive judgments. We first looked for the evidence of “meta-metacognitive ability” by examining the difference in metacognitive efficiency between trials with high and low Type-3 ratings. Then, we investigated whether similar behavioral results emerge between a condition where a binary Type-2 rating is followed by a binary Type-3 rating (Type-2/Type-3 condition) and a condition where a single Type-2 rating is provided on a 4-point scale (Type-2-only condition).

### 3.1 Equivalent meta-metacognitive performance in Type-2/Type-3 and Type-2-only conditions

We first investigated whether participants demonstrated positive meta-metacognitive (Type-3) ability in the Type-2/Type-3 condition. To do so, we compared the Mratio values based on the Type-1 and Type-2 responses separately for trials in which the Type-3 rating was high vs. low. To control for the influence of outliers, for this analysis, we excluded two participants who had extreme Mratio values (5.43 and −11.74) in the Type-2-only condition.

**Figure 3.**
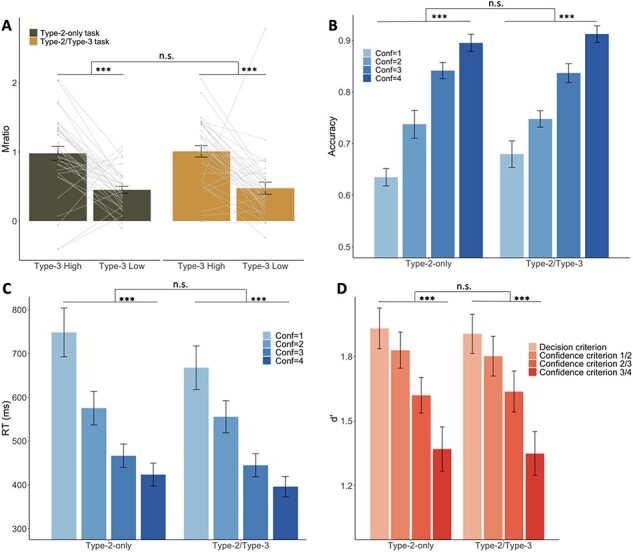
Equivalent results in Type-2/Type-3 and Type-2-only conditions. (a) We found higher metacognitive efficiency (Mratio) in trials with high vs. low Type-3 responses. Critically, equivalent results were obtained in both the Type-2/Type-3 condition and the Type-2-only condition (after rating conversion where the 4-point Type-2 ratings are turned into a type-3 rating combination). (b) Accuracy associated with each confidence rating in the Type-2-only condition and the converted ratings in the Type-2/Type-3 condition. Accuracy increased with confidence to the same extent in both conditions. (c) RT associated with each confidence rating in the Type-2-only condition and the converted ratings in the Type-2/Type-3 condition. RT decreased with confidence to the same extent in both conditions. (d) Sensitivity (*d*') associated with each decision and confidence criterion. Sensitivity decreases for criteria further away from the decision criterion, as expected from the existence of signal-dependent metacognitive noise (Shekhar and Rahnev 2021b). Critically, the decrease is equivalent for the Type-2-only and the Type-2/Type-3 conditions, indicating the absence of additional “meta-metacognitive noise” that may be expected from a separate Type-3 system. Here, “confidence criterion *n* /*n* + 1” indicates the confidence criterion that separates the ratings *n* and *n* + 1. n.s., not significant; ***, *P* < .001

We found that high Type-3 ratings were associated with significantly higher Mratio values (mean = 0.96) than low Type-3 ratings [mean = 0.46; *t*(35) = 4.39, *P* < .001; Fig. 3a, right panel]. This result indicates that Type-3 ratings were meaningful and could be used to determine whether the Type-2 rating was more or less appropriate.

Critically, we examined whether a similar effect would emerge in the Type-2-only condition. To perform the equivalent analysis in that condition, we first converted participants’ Type-2 confidence ratings given on a 4-point scale into binary Type-2 and binary pseudo-Type-3 ratings (Fig. 1c). Specifically, the Type-2 ratings 1, 2, 3, and 4 were converted into {low, high}, {low, low}, {high, low}, and {high, high} where the first variable indicates the Type-2 and the second variable indicates the pseudo-Type-3 rating after the conversion. We then performed the equivalent analysis as earlier. We found virtually identical results with what we observed in the Type-2/Type-3 condition. Specifically, for the converted ratings, high Type-3 ratings were again associated with significantly higher Mratio values (mean = 0.98) than low Type-3 ratings [mean = 0.45; *t*(33) = 4.90, *P* < .001; Fig. 3a, Fig. 3a, left panel] and there was no interaction between the results for the Type-2/Type-3 and the Type-2-only conditions [*F*(1132) = 0.001, *P* = .976]. Furthermore, a Bayesian paired-sample *t*-test revealed that the Mratio difference between high and low pseudo-Type-3 ratings in the Type-2-only condition (mean = 0.527) did not differ from the same difference in the Type-2/Type-3 condition (mean = 0.532; Bayes Factor (BF)_01_ = 5.439). Moreover, we additionally conducted linear LLM analysis with participant as the random effect and found a main effect of Type-3 certainty on Mratio (*P *< .001), but no effect of task condition (*P *= .755) and no interaction between the task condition and Type-3 certainty (*P* = .976). Together, these results demonstrate that the Type-2/Type-3 and Type-2-only conditions produced equivalent “meta-metacognitive” effects.

### 3.2 Equivalent accuracy and RT in Type-2/Type-3 and Type-2-only conditions

To further assess the similarity between the two conditions, we examined the Type-1 accuracy and Type-1 decision RT associated with each rating in each condition. For ease of comparison, we performed the opposite conversion compared to the aforementioned analysis. Specifically, we converted the ratings from the Type-2/Type-3 condition into the 4-point scale used in the Type-2-only condition (Fig. 1c). We then computed the Type-1 accuracy and average Type-1 decision RT for each confidence rating and compared these across the two conditions.

We found that Type-1 accuracy increased for higher confidence ratings and that this effect was similar across the two conditions (Fig. 3b). Indeed, results from a linear LLM with participant as a random effect demonstrated a significant main effect of the Type-2 confidence level on Type-1 decision accuracy (*P* < .001), but no main effect of the task condition (*P* = .139) and no interaction between Type-2 confidence and task condition (*P* = .317). Post hoc *t*-tests further confirmed that there was no difference in Type-1 accuracy between the two conditions for each of the four Type-2 confidence levels [confidence = 1: *t*(55.655) = 1.460, *P* = .150, BF_01_ = 1.586; confidence = 2: *t*(55.226) = 0.328, *P* = .744, BF_01_ = 3.903; confidence = 3: *t*(68.494) = −0.202, *P* = .841, BF_01_ = 4.043; confidence = 4: *t*(68.926) = 0.734, *P* = .465, BF_01_ = 3.246].

Similar to Type-1 accuracy, we found that Type-1 decision RT decreased for higher confidence ratings with this effect again being similar across the two conditions (Fig. 3c). Again, results from a linear LLM with participant as a random effect confirmed that there was a main effect of the Type-2 confidence level on Type-1 decision RT (*P* < .001), but no main effect of task condition (*P* = .106) and no interaction between Type-2 confidence and task condition (*P* = .368). Post hoc *t*-tests revealed that there was no difference in Type-1 decision RT between two conditions for each of the four Type-2 confidence levels [confidence = 1: *t*(66.707) = −1.081, *P* = .284, BF_01_ = 2.470; confidence = 2: *t*(68.758) = −0.370, *P* = .713, BF_01_ = 3.856; confidence = 3: *t*(69.974) = −0.576, *P* = .566, BF_01_ = 3.567; confidence = 4: *t*(68.253) = −0.784, *P* = .437, BF_01_ = 3.145]. Thus, equivalent results emerged for both accuracy and RT in the Type-2/Type-3 and Type-2-only conditions.

### 3.3 Equivalent levels of noise in Type-2/Type-3 and Type-2-only conditions

One of the foundational properties of Type-2 metacognitive ratings of confidence is that they are noisier than Type-1 object-level decisions, a phenomenon termed metacognitive inefficiency (Shekhar and Rahnev 2021a). Computationally, such inefficiency has often been modeled as metacognitive noise, i.e. noise that affects Type-2 but not Type-1 ratings (Barrett et al. 2013, Maniscalco and Lau 2016, Fleming and Daw 2017, Bang et al. 2019, Xue et al. 2021, Shekhar and Rahnev 2021b). As demonstrated by Shekhar and Rahnev (2021a), metacognitive noise expresses itself in the phenomenon where perceptual sensitivity (*d*') is lower when it is computed based on one of the confidence criteria rather than based on the decision criterion. If the Type-3 judgments are produced by a separate system compared to Type-2 judgments, that may be reflected in the existence of “meta-metacognitive noise” associated with Type-3 ratings. Such meta-metacognitive noise would result in an additional drop in the sensitivity (*d*') estimated based on confidence criteria in the Type-2/Type-3 compared to the Type-2-only condition.

To check for such an effect, we computed *d*' separately based on (i) the decision criterion, (ii) the criterion separating confidence Levels 1 and 2, (iii) the criterion separating confidence Levels 2 and 3, and (iv) the criterion separating confidence Levels 3 and 4. We did that separately for the Type-2-only condition and for the converted ratings from the Type-2/Type-3 condition. We found no difference in *d*' for any of the criteria (Fig. 3d). Indeed, there was no significant difference in *d*' levels between the two conditions for the decision criterion [*t*(69.887) = 0.197, *P* = .844, BF_01_ = 5.001], the confidence criterion separating ratings 1 and 2 [*t*(69.382) = 0.226, *P* = .822, BF_01_ = 4.782], the confidence criterion separating ratings 2 and 3 [*t*(68.62) = −0.137, *P* = .891, BF_01_ = 5.331], or the confidence criterion separating ratings 3 and 4 [*t*(69.986) = 0.142, *P* = .887, BF_01_ = 5.394]. The *d*' decreased monotonically for confidence criteria further separated from the decision criterion in both the Type-2-only (Pearson’s *r* = -0.363, *P* < .001) and Type-2/Type-3 conditions (Pearson’s *r* = −0.341, *P* < .001). Critically, a Bayesian correlation equality test revealed that the degree of *d*' decrease was equivalent between the two conditions (BF_01_ = 10.084). We also confirmed the aforementioned results using a linear LLM, which demonstrated a significant main effect of the distance between the confidence criterion and the decision criterion on *d*' (*P* < .001), but no effect of task condition (*P* = .692) and no interaction between the location of the confidence criterion and the task condition (*P *= .822). Thus, these results replicate previous findings that metacognitive noise is signal-dependent (i.e. it increases away from the decision criterion; Shekhar and Rahnev 2021a) and suggest that there is no extra noise in the Type-3 computation compared to an equivalent Type-2 computation.

### 3.4 Limited influence of task order on the observed behavioral similarities

The aforementioned results show that the Type-2-only and Type-2/Type-3 conditions produced equivalent results, suggesting the existence of common computations for metacognition and meta-metacognition. One possible concern with this conclusion is that participants may have used the binary Type-2 and Type-3 scales to express their confidence in a single 4-point scale. If this is the case, the observed similarity between Type-2 and Type-3 processes should be stronger among participants who first completed the Type-2-only condition and were thus already familiar with the 4-point scale by the time they saw the Type-2/Type-3 condition.

To check for this possibility, we investigated how task order affected the behavioral indexes of interests. Specifically, we examined how task order modulated the effects we observed on Mratio (Fig. 3a), Type-1 accuracy (Fig. 3b), Type-1 decision RT (Fig. 3c), and estimated *d*' using different confidence criteria (Fig. 3d). First, a linear LLM revealed that task order did not moderate the effect of Type-3 certainty on Mratio [*F*(1,34) = 0.183, *P* = .672]. Second, a similar LLM showed that task order did not influence the relationship between the Type-2 confidence level and Type-1 decision accuracy [*F*(1, 247.56) = 0.037, *P* = .848]. Third, another LLM revealed a significant but small 3-way interaction between task order × task condition × Type-2 level on Type-1 decision RT [*F*(1, 247.56) = 4.361, *P* = .039]. However, this three-way interaction reflects the fact that Type-1 decision RTs generally decrease over time, so the Type-2-only condition produces overall slower RTs when it comes first and overall faster RTs when it comes second. Finally, another LLM revealed no effect of task order on the relationship between the distance of each confidence criterion to the decision criterion and the sensitivity (*d*') associated with that confidence criterion [*F*(1, 252) = 1.520, *P* = .210]. Taken together, these results indicate that having experienced Type-2 confidence in the 4-point scale first did not evoke higher similarities between the Type-2/Type-3 and the Type-2-only conditions, suggesting that our participants were unlikely to use the binary Type-2 and Type-3 scales to express their confidence in a single 4-point scale in the Type-2/Type-3 condition.

## 4 Discussion

We examined whether humans are likely to have different systems for metacognition and meta-metacognition in the context of perception. We asked participants to perform a simple perceptual decision-making task where they gave either a Type-2 metacognitive judgment on a 4-point scale or a combination of binary Type-2 metacognitive judgment and a binary Type-3 meta-metacognitive judgment. We found equivalent performance between the two conditions in terms of metacognitive efficiency, Type-1 accuracy, Type-1 decision RT, and metacognitive noise. These results suggest that there might be no fundamental difference between perceptual metacognition and meta-metacognition such that the two types of judgments may be made by the same unified system.

Human meta-metacognitive ability seems intuitive. For instance, Händel and Fritzsche (2016) reported that low-performing students not only overestimated their performance but also knew that they had low ability to estimate their performance. Recht et al. (2022) recently empirically demonstrated the existence of meta-metacognition. The researchers asked participants to compare two consecutive Type-2 ratings at the Type-3 level and even rate the certainty in the Type-3 rating at the Type-4 level. They found evidence for up to fourth-level metacognitive ability, although they could not demonstrate Mratio differences for low vs. high Type-3 ratings. Using a similar paradigm, Sherman and Seth (2023) also observed evidence for Type-3 meta-metacognitive ability, but again did not find an Mratio difference between low and high Type-3 ratings. By using a slightly different task, we confirmed these recent findings of meta-metacognitive ability and further extended them by demonstrating a significant Mratio difference between high and low Type-3 judgments.

Despite being able to recursively evaluate lower-order Type-1 decisions, it is still unclear how people computationally implement these higher-order metacognitive processes. There is still controversy about whether Type-1 cognition and Type-2 metacognition are produced by a unified system or two distinct systems (Nelson and Narens 1990, Pleskac and Busemeyer 2010, Fleming and Daw 2017, Mamassian 2020, Desender et al. 2021). Although the current study cannot clearly resolve this controversy, our results point toward a common computation between metacognition and meta-metacognition and thereby highlight two hypotheses on the human cognitive architecture. First, it is possible that there are two separate cognitive systems: a lower-level system specialized for Type-1 judgments and a higher-level system that is responsible for self-evaluations of these Type-1 judgments, encompassing both metacognitive and meta-metacognitive aspects. Alternatively, a more parsimonious hypothesis is that there is a unified system governing both lower-level Type-1 decisions and all higher-level self-evaluations. Future research is needed to examine the similarities between cognition and metacognition and thereby provide a more precise picture of human cognitive architecture.

Although here we argued that there is a single system producing Type-2 and Type-3 judgments, there could be at least two counter-arguments against this conclusion. First, it is possible that there are separate Type-2 and Type-3 systems, but participants simply did not engage the Type-3 system in the current experiment. In that interpretation, participants simply used the combination of binary Type-2 and binary Type-3 scales as a single 4-point Type-2 scale. Our task order analyses showed that this interpretation is unlikely because all effects were observed equally for participants who first experience the Type-2/Type-3 condition and participants who first experienced the Type-2-only condition. Second, it could be argued that separate Type-2 and Type-3 systems exist (and that participants actively engaged the Type-3 system), but that the two systems happened to perform the same computations in the current task. This possibility appears unlikely but is hard to disprove. Ultimately, our data cannot completely falsify either possibility. Indeed, it is difficult to prove that something (e.g. a separate Type-3 system) does not exist since this would be akin to proving the null hypothesis. The final conclusion on this issue would require convergent evidence across many tasks and laboratories over time. Nevertheless, given the current evidence, we argue that a common system for Type-2 and Type-3 judgments is the most parsimonious hypothesis.

It should be noted that our conclusions are specific to the perceptual domain and it remains an open question to what extent they generalize to other domains. For example, it may be that in the context of more complex judgments such as theory of mind (Proust 2007, Carruthers 2009), at least partially separable systems exist for progressively higher-level metacognitive judgments. Given current controversies regarding whether metacognition is domain-general or domain-specific (McCurdy et al. 2013, Lee et al. 2018, Morales et al. 2018, Mazancieux et al. 2020, Zheng et al. 2021), it remains possible that our conclusions in the context of perception not fully apply to other domains. Future research should examine to what extent that current results on the perceptual meta-metacognition generalize to other domains like memory or theory of mind.

## Conclusion

In summary, the current study confirmed that people have the meta-metacognitive ability to evaluate the appropriateness of their metacognitive judgments. Critically, our findings suggest that a single system produces perceptual metacognitive and meta-metacognition decisions. These results cast doubts on whether there are different systems for cognition and metacognition in the context of perception.

## Supplementary Material

niad023_Supp

## Data Availability

All data and corresponding analysis code underlying this article are available at https://osf.io/n856x/.

## References

[R1] Bahrami B, Olsen K, Latham PE et al. Optimally interacting minds. *Science* 2010;329:1081–5. 20798320 10.1126/science.1185718 PMC3371582

[R2] Bang JW, Shekhar M, Rahnev D. Sensory noise increases metacognitive efficiency. *J Exp Psychol Gen* 2019;148:437–52. 30382720 10.1037/xge0000511

[R3] Barrett AB, Dienes Z, Seth AK Measures of metacognition on signal-detection theoretic models. *Psychol Methods* 2013;18:535–52. 24079931 10.1037/a0033268

[R4] Carruthers P . Mindreading underlies metacognition. *Behav Brain Sci* 2009;32:164–82. 10.1017/S0140525X09000545 19386144

[R5] de Leeuw JR . jsPsych: A JavaScript library for creating behavioral experiments in a Web browser. *Behav Res* 2015;47:1–12. 10.3758/s13428-014-0458-y 24683129

[R6] Desender K, Boldt A, Yeung N. Subjective confidence predicts information seeking in decision making. *Psychol Sci* 2018;29:761–78. 29608411 10.1177/0956797617744771

[R7] Desender K, Ridderinkhof KR, Murphy PR. Understanding neural signals of post-decisional performance monitoring: an integrative review. *ELife* 2021;10:e67556. 10.7554/eLife.67556 PMC8378845 34414883

[R8] Fleming SM, Daw ND. Self-evaluation of decision-making: a general Bayesian framework for metacognitive computation. *Psychol Rev* 2017;124:91–114. 28004960 10.1037/rev0000045 PMC5178868

[R9] Fleming SM, Weil RS, Nagy Z et al. Relating introspective accuracy to individual differences in brain structure. *Science* 2010;329:1541–3. 20847276 10.1126/science.1191883 PMC3173849

[R10] Gilbert SJ, Bird A, Carpenter JM et al. Optimal use of reminders: metacognition, effort, and cognitive offloading. *J Exp Psychol Gen* 2020;149:501–17. 31448938 10.1037/xge0000652

[R11] Green DM, Swets JA. *Signal Detection Theory and Psychophysics*. New York: Wiley, 1966.

[R12] Guggenmos M, Wilbertz G, Hebart MN et al. Mesolimbic confidence signals guide perceptual learning in the absence of external feedback. *ELife* 2016;5:e13388. 10.7554/eLife.13388 PMC4821804 27021283

[R13] Händel M, Fritzsche ES Unskilled but subjectively aware: metacognitive monitoring ability and respective awareness in low-performing students. *Mem Cognit* 2016;44:229–41. 10.3758/s13421-015-0552-0 26438233

[R14] Kiani R, Shadlen MN Representation of confidence associated with a decision by neurons in the parietal cortex. *Science* 2009;324:759–64. 19423820 10.1126/science.1169405 PMC2738936

[R15] Lee ALF, Ruby E, Giles N et al. Cross-domain association in metacognitive efficiency depends on first-order task types. *Front Psychol* 2018;9:2464. 10.3389/fpsyg.2018.02464 PMC6288301 30564183

[R16] Macmillan NA, Creelman CD. *Detection Theory: A User’s Guide*. New York: Psychology Press, 2004.

[R17] Mamassian P . Confidence forced-choice andother metaperceptual tasks. *Perception* 2020;49:616–35. 32552488 10.1177/0301006620928010

[R18] Maniscalco B, Lau H. A signal detection theoretic approach for estimating metacognitive sensitivity from confidence ratings. *Conscious Cogn* 2012;21:422–30. 22071269 10.1016/j.concog.2011.09.021

[R19] Maniscalco B, Lau H. The signal processing architecture underlying subjective reports of sensory awareness. *Neurosci Conscious* 2016;2016:niw002. 10.1093/nc/niw002 PMC4972343 27499929

[R20] Mazancieux A, Fleming SM, Souchay C et al. Is there a G factor for metacognition? Correlations in retrospective metacognitive sensitivity across tasks. *J Exp Psychol Gen* 2020;149:1788–99. 32191079 10.1037/xge0000746 PMC7397761

[R21] McCurdy LY, Maniscalco B, Metcalfe J et al. Anatomical coupling between distinct metacognitive systems for memory and visual perception. *J Neurosci* 2013;33:1897–906. 23365229 10.1523/JNEUROSCI.1890-12.2013 PMC4696871

[R22] Morales J, Lau H, Fleming SM. Domain-general and domain-specific patterns of activity supporting metacognition in human prefrontal cortex. *J Neurosci* 2018;38:3534–46. 29519851 10.1523/JNEUROSCI.2360-17.2018 PMC5895040

[R23] Nelson TO Narens L . Metamemory: a theoretical framework and some new findings. In: Bower GH (ed.), *Psychology of Learning and Motivation*. Vol. 26, New York: Academic Press, 1990, 125–73.

[R24] Pescetelli N, Yeung N. The role of decision confidence in advice-taking and trust formation. *J Exp Psychol Gen* 2021;150:507–26. 33001684 10.1037/xge0000960

[R25] Pleskac TJ, Busemeyer JR. Two-stage dynamic signal detection: a theory of choice, decision time, and confidence. *Psychol Rev* 2010;117:864–901. 20658856 10.1037/a0019737

[R26] Pouget A, Drugowitsch J, Kepecs A. Confidence and certainty: distinct probabilistic quantities for different goals. *Nat Neurosci* 2016;19:366–74. 26906503 10.1038/nn.4240 PMC5378479

[R27] Proust J . Metacognition and metarepresentation: is a self-directed theory of mind a precondition for metacognition? *Synthese* 2007;159:271–95.

[R28] Rahnev D, Bahdo L, de Lange FP et al. Prestimulus hemodynamic activity in dorsal attention network is negatively associated with decision confidence in visual perception. *J Neurophysiol* 2012;108:1529–36. 22723670 10.1152/jn.00184.2012

[R29] Ratcliff R, Starns JJ. Modeling confidence judgments, response times, and multiple choices in decision making: recognition memory and motion discrimination. *Psychol Rev* 2013;120:697–719. 23915088 10.1037/a0033152 PMC4106127

[R30] Recht S, Jovanovic L, Mamassian P et al. Confidence at the limits of human nested cognition. *Neurosci Conscious* 2022;2022:niac014. 10.1093/nc/niac014 PMC9574785 36267224

[R31] Rouault M, Seow T, Gillan CM et al. Psychiatric symptom dimensions are associated with dissociable shifts in metacognition but not task performance. *Biol Psychiatry* 2018;84:443–51. 29458997 10.1016/j.biopsych.2017.12.017 PMC6117452

[R32] Seow TXF, Rouault M, Gillan CM et al. How local and global metacognition shape mental health. *Biol Psychiatry* 2021;90:436–46. 34334187 10.1016/j.biopsych.2021.05.013

[R33] Shea N, Boldt A, Bang D et al. Supra-personal cognitive control and metacognition. *Trends Cogn Sci* 2014;18:186–93. 24582436 10.1016/j.tics.2014.01.006 PMC3989995

[R34] Shekhar M, Rahnev D. Distinguishing the roles of dorsolateral and anterior pfc in visual metacognition. *The Journal of Neuroscience: The Official Journal of the Society for Neuroscience* 2018;38:5078–87. 29720553 10.1523/JNEUROSCI.3484-17.2018 PMC6705938

[R35] Shekhar M, Rahnev D. Sources of metacognitive inefficiency. *Trends Cogn Sci* 2021a;25:12–23. 33214066 10.1016/j.tics.2020.10.007 PMC8610081

[R36] Shekhar M, Rahnev D. The nature of metacognitive inefficiency in perceptual decision making. *Psychol Rev* 2021b;128:45–70. 32673034 10.1037/rev0000249 PMC7883626

[R37] Sherman M, Seth A. Knowing that you know that you know: Above chance discrimination of metacognitive performance. PsyArXiv 2023. 10.1093/nc/niae020 PMC11110933 38779689

[R38] Xue K, Shekhar M, Rahnev D. Examining the robustness of the relationship between metacognitive efficiency and metacognitive bias. *Conscious Cogn* 2021;95:103196. 10.1016/j.concog.2021.103196 PMC8560567 34481178

[R39] Zalla T, Miele D, Leboyer M et al. Metacognition of agency and theory of mind in adults with high functioning autism. *Conscious Cogn* 2015;31:126–38. 25482271 10.1016/j.concog.2014.11.001

[R40] Zheng Y, Wang L, Gerlofs DJ et al. Atypical meta-memory evaluation strategy in schizophrenia patients. *Schizophr Res Cogn* 2022;27:100220. 10.1016/j.scog.2021.100220 PMC8501761 34646754

[R41] Zheng Y, Wang D, Ye Q et al. Diffusion property and functional connectivity of superior longitudinal fasciculus underpin human metacognition. *Neuropsychologia* 2021;156:107847. 10.1016/j.neuropsychologia.2021.107847 33812946

