# Supplementary figures and images for: Common computations for metacognition and meta-metacognition
Source: Neurosci Conscious. 2023 Nov 7;2023(1):niad023. doi: 10.1093/nc/niad023 (PMC10693288; doi:10.1093/nc/niad023)

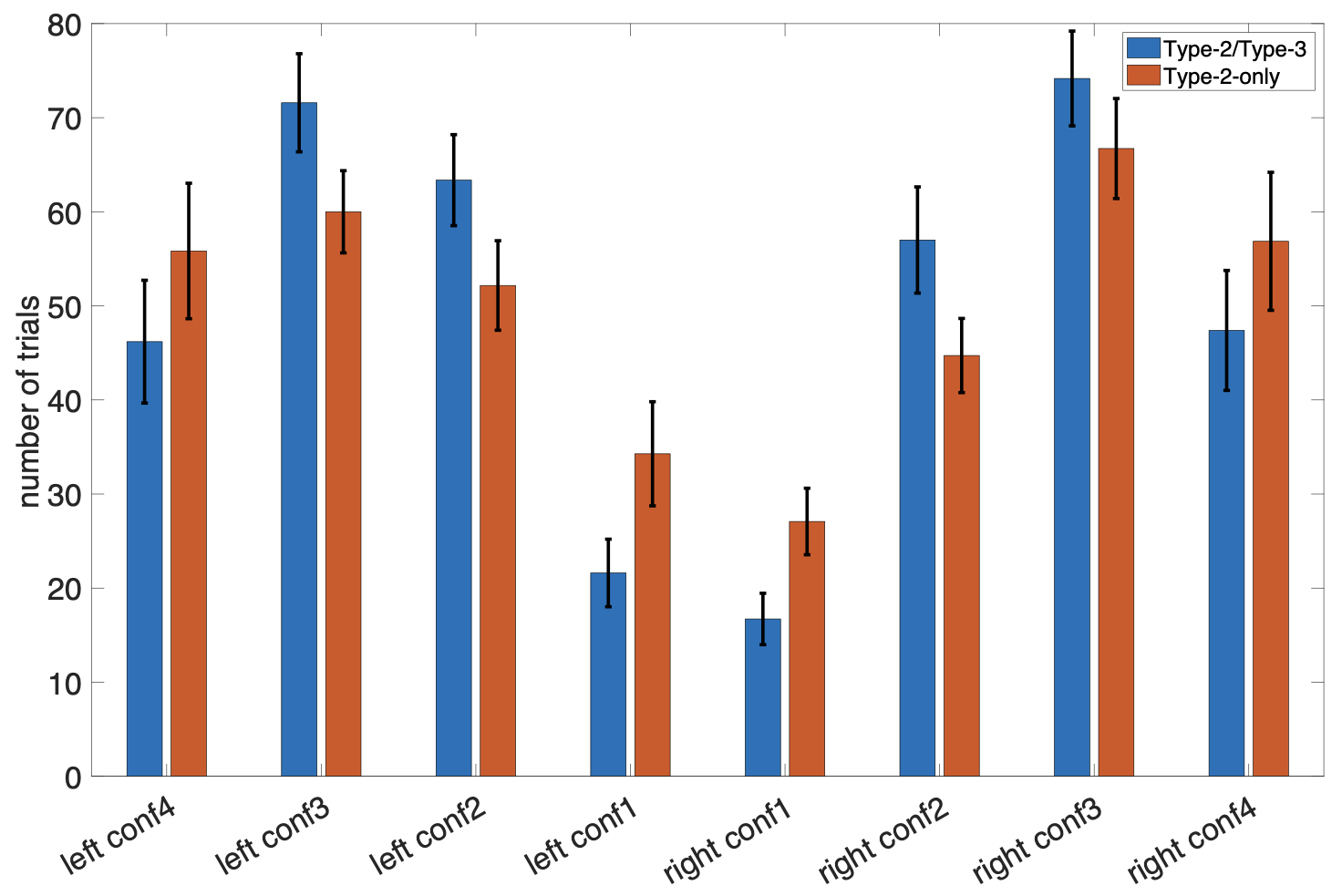

Supplement: niad023_Supp [file niad023_supp.zip › Figure S1.tiff]
